# Metabolic Reprogramming, Questioning, and Implications for Cancer

**DOI:** 10.3390/biology10020129

**Published:** 2021-02-07

**Authors:** Pierre Jacquet, Angélique Stéphanou

**Affiliations:** Université Grenoble Alpes, CNRS, TIMC/BCM, 38000 Grenoble, France; pierre.jacquet1@univ-grenoble-alpes.fr

**Keywords:** metabolic reprogramming, Warburg effect, cancer metabolism

## Abstract

**Simple Summary:**

The right terminology to describe biological phenomena is important. An inapropriate use can create some bias in the understanding that are not without consequences. In this communication we focus on the use of the term “reprogramming” associated to cancer cell metabolism.

**Abstract:**

The expression “metabolic reprogramming” has been encountered more and more in the literature since the mid-1990s. It seems to encompass several notions depending on the author, but the lack of a clear definition allows it to be used as a “catch-all” expression. Our first intention is to point out the inconsistencies in the use of the reprogramming terminology for cancer metabolism. The second is to address the over-focus of the role of mutations in metabolic adaptation. With the increased interest in metabolism and, more specifically, in the Warburg effect in cancer research, it seems appropriate to discuss this terminology and related concepts in detail.

## 1. Introduction

In recent publications, some authors have defined metabolic reprogramming as the “changes of tumor cellular bioenergetics” [1]; “phenomenon that cancer cells reprogram some of their metabolisms” [2]; “the ability of cancer cells to alter their metabolism in order to support the increased energy request[...]” [3] or “mechanism by which cells rewire their metabolism to promote proliferation and cell growth” [4]. These definitions are based, in particular, on the observation that significant areas in tumors exhibit lactic acidosis as well as increased glucose consumption (commonly called the Warburg effect). These changes mainly affect the production of cell energy (in the form of ATP), the synthesis of amino acids, and the surrounding microenvironment.

These definitions also suggest that cancer cells drive their metabolism into a “non-physiological” state in order to fulfill their needs, but the underlying mechanism is not very clear. This anthropomorphism applied to the cell is frequently encountered in such definitions. This makes the theoretical concepts applied to the cell drift towards deterministic mechanisms, which is not necessarily true. Twenty-five years ago, “metabolic reprogramming” used to be related to a genetic/epigenetic origin. It can be tempting to associate genetic modifications and “reprogramming” because of the computational culture in science and, more recently, in biology. However, if there is “reprogramming”, the cell should contain a modified program, implying both the existence of a program and an alternative version of this program. The concept of “program” in biology is often debated and does not reach consensus. The problem is linked to the long-discussed “cell-machine” metaphor [5,6]. A good review on this subject and on the risk of using metaphors in biological sciences was made by Boudry and Pigliucci [7]. The issue that we are addressing here is not only the formulation of a “semantic” problem, but a larger reflection on the perception of cancer metabolism in the literature. The idea of “reprogramming”, beyond its semantic problematic, is linked to the concept of change in gene expression and metabolic routing. It is therefore interesting to see whether cancer cells must necessarily exhibit a radically different gene expression profile or particular structural modifications to adopt a different metabolism. In this paper, we address one consequence of this semantic drift, which is that cancer cell metabolism is almost systematically described as different from that of a normal cell, and this might not necessarily always be the case.

The two questions that underline our reasoning are: Is it possible to identify one unique universal metabolic feature of cancer cells despite tumor heterogeneity? Can we consider the adaptability of the cell as the main factor in metabolic transitions beyond pathological processes?

## 2. Is There One Metabolic Signature That Distinguishes a Normal and Tumor Phenotype?

The question that arises is whether metabolic features of cancers are sufficient to portray two different metabolisms (i.e., one healthy and one pathological). For years, many articles [8,9,10] have emphasized the dual nature of cancer cells: the metabolic switch, for example, is often used to designate the transition from a metabolism based on oxidative phosphorylation to glycolysis, and it often refers to the Warburg effect. The Warburg effect is the observation made by Otto Warburg and published in 1956, stating that cancer cells produce a lot of lactate and consume an elevated amount of glucose. It is the fermentation where the glucose transformed into pyruvate is directed to lactate production. What makes this process peculiar is when it occurs in the presence of oxygen (aerobic glycolysis). Fermentation occurs generally in hypoxia, while cells use respiration in the presence of oxygen. Some authors have pointed out that the use of the term “metabolic switch” might be inappropriate and confusing, and question its nature [11,12]. Indeed, it is now established that cancer cells use both modes of energy production (fermentation and respiration) simultaneously, but in different proportions [13]. The observation that cells can use fermentation with oxygen does not necessarily imply that oxygen is always available or that glucose is their only substrate. The main idea expressed by Warburg in his article “On the origin of cancer cells” [14] was that of the over-expression of fermentation before considering the presence of oxygen or lack thereof. Consequently, this includes both aerobic and anaerobic glycolysis. However, the Warburg effect is today viewed as aerobic glycolysis only; anaerobic glycolysis, which refers to the well-known Pasteur effect, is not cancer specific. Progressively, Warburg’s observation was reduced to aerobic glycolysis, and more and more in the post-1990s literature. Furthermore, some cells lines show strong differences between glucose uptake and lactate secretion, strongly suggesting a non-Warburgian phenotype [11]. In fact, labeling cancer metabolism as “aerobic glycolytic” or described by a “metabolic switch” [13] hides many different metabolic states and the possibility of transient states, and creates a dichotomy in which there are only two exclusive modes of operation for the cell. The prevalence of certain conditions does not exclude the existence of others, which, if they do exist, cannot simply be considered as artifacts.

A recent study [8] showed that under lactic acidosis (a condition encountered in most solid tumors), cells gradually abandon the Warburg phenotype to revert to a non-glycolytic phenotype (high ratio of oxygen consumption). In lactic acidosis, the intracellular pH is lower, which tends to inhibit the activity of most glycolytic enzymes. Protons and lactate, both products of fermentation, are themselves responsible for its alteration. It is a form of feedback inhibition of the glycolysis. The authors of [8] proposed that the Warburg effect confers growth advantage until there is not enough glucose or lactic acid; then, cells need to adapt to the microenvironment and use other substrates. Moreover, extracellular lactate is a substrate that can be reused by the cell, converted back to pyruvate, and used in the tricarboxylic acid (TCA) cycle, a fact that is often overlooked. Thus, the Warburg effect is not always seen in each cancer cell of a given tumor. In these conditions, the Warburg effect cannot be seen as a universal/ubiquitous metabolic marker of cancer cells. It may, however, represent a good marker at the tissue scale. Multiple techniques use the metabolism to target cancer cells and tissues. In particular, FDG-PET (Fluorodeoxyglucose Positron Emission Tomography) scanning is used for cancer diagnosis through the detection of high-glucose-uptake areas. However, this identification by the use of radiopharmaceutical analogues of glucose alone does not ensure the perfect detection of all cancer cells in all cancer types [1,15].

In addition, the metabolic orientation of cells towards the use of aerobic glycolysis is not specific to cancer. Aerobic glycolysis can be frequently encountered during brain activation [16], vessel sprouting, or the development of trained immunity [17], since it is the fastest way to provide ATP for such acute demands. We can thus wonder if the focus of recent years on aerobic glycolysis as a “hallmark of cancer” is not symptomatic of the lack of attention paid to heterogeneity as well as the difficulty of integrating it into models.

Several studies have shown that the metabolic heterogeneity within tumor cells of the same tissue was an obstacle to the identification of a common metabolic profile in all these cells [13,15]. Moreover, the overall/bulk profile of these tissues remains similar to that of their non-transformed version [18]. If it was possible to predict the tumorigenicity of a cell by its metabolism alone, it would likely be done by considering multiple converging factors rather than just one. Finding a universal target for cancer metabolism would be a huge leap in the search for a therapeutic solution. However, for now, this universal target of metabolism, if it exists, is not yet known, although there may be promising leads, such as a reversed intra/extracellular pH gradient [19,20,21,22,23].

## 3. How Important Is the Influence of the Tumor Heterogeneity on the Metabolism at the Tissue Scale?

The tumor and environmental heterogeneities have raised increasing interest. The environmental heterogeneity is responsible for many metabolic transitions (lactic acidosis is one example, but hypoxia or nutrient starvation are also often encountered in the most central parts of the tumors). Many theoretical and experimental models try to address the complexity of cancer metabolism, but face difficulty in grasp the huge heterogeneity of cells and microenvironmental conditions. It is important to be cautious about the conclusions drawn from models based on cell populations that are too homogeneous and on global measurements at the tissue scale [24].

Technological advances in the measurement of metabolic parameters at the cell level made possible the identification of very heterogeneous behaviors [13] depending on the cell location in the same tissue and revealed subtle dynamics that do not appear at the whole-tissue scale [25]. This has been confirmed in a study [13] on two cell lines (melanoma and Head and neck squamous cell carcinoma (HNSCC)) where single-cell RNA-seq and bulk RNA-seq were compared and showed big differences. In fact, bulk measurements tend to mask differences between cells by averaging expression levels. In this study, variation in oxidative phosphorylation (OXPHOS) activity is the main contributor to the metabolic heterogeneity in both malignant and normal cells and serves as an oxygen availability sensor while stabilizing hypoxia-induced factors (HIFs). The tumor heterogeneity also includes mechanisms of supposed/apparent cooperation between tumor cells, as well as with stromal cells (CAFs) [26], some producing lactate by fermentation, lactate captured and used by others under different extra-cellular conditions, which is named the “reverse Warburg effect” or “two-compartment metabolic coupling”. Temporal heterogeneity is also important. Cells are subjected to variations in oxygenation due to angiogenesis. They occur in the form of cycles, following vascular maturation [27,28].

The quantification of the respective contributions to the metabolism of genetic/ epigenetic factors on the one hand and biophysical factors imposed by the environment on the other hand is technically very difficult to obtain. This technical difficulty comes from the lack of data and data heterogeneity, since these two factors are rarely measured together. It is quite possible to imagine that their proportions vary greatly between the tissues from which the tumors originate and from one person to another.

## 4. Reprogramming or Adaptability?

Let us assume that there is a clearly established program of metabolism by making the analogy with a computer program (i.e., a sequence of instructions that define the entire behavior of a system). In that case, three solutions emerge:

1. The different metabolic scenarios are already part of the “program” as conditional blocks (if–else blocks); therefore, there is no “reprogramming”.
2. Parts of the code are randomly added or deleted, such as random mutations more akin to gain/loss of functions. This is following a classic evolutionary mechanism and not an in-depth transformation of the program itself.
3. The program and its structure are changed deterministically in an optimal way and in a single attempt (copy–paste). Such determinism that evades trials and errors processes is not documented in biology.

It is interesting to note that the first hypothesis belongs to dynamical systems that are predefined by laws and rules. As such, they are fully deterministic. If the cell is a dynamical system, then the program is pre-existing, and therefore, there is no reprogramming, since the structure of the system is unchanged. All the metabolic behaviors in our case are thus accounted for, and they spontaneously emerge according to the external constraints under the form of stable or unstable attractors (including chaotic states).

In opposition to the dynamical systems, the cell can be described as an entity without predefined rules, and therefore, it constantly evolves in the Darwinian sense (second hypothesis). This means that the cell is constitutively unstable (the rules can change), and therefore, it is a non-deterministic system. It functions through trial and error and reaches probabilistic optimality through the law of large numbers under the pressure of the environment.

Is the program of a cancer cell essentially different from that of a normal cell [13]? How many of the changes observed in cancer can be attributed to mutations, altered gene expression, or new routes in metabolic networks, and how many can be attributed to a “natural” change of state inside the metabolic landscape in response to external factors (as in the dynamical system sense)?

It is now well established that cancers all present mutations and modifications of gene expression. The TP53 gene, in particular, which is one of the most frequently mutated genes in tumors, affects the metabolism by inhibiting, in normal cells, the transcription of glucose transporters (Glut1 and Glut4) [29]. The mutation of this gene removes a barrier against a rise in Glut expression found in cancer cells. Many other genes have their expression modulated in cancer, with multiple repercussions on the metabolism: HIF-1α affects the response to low oxygen concentration [30], and c-Myc enhances cell proliferation, thus increasing metabolic needs [31].

Are genetic mutations the drivers of metabolic modifications of the cell and, thereby, transform the environment? Alternatively, is it the other way around [32], or both? Recently, 28 lysine lactylation sites on histones (Kla) have been identified [33], showing that increased lactate concentration leads to modification of Kla and its associated gene expression. Low pH [34] and surrounding cells subjected to chronic inflammation [35] are known to promote carcinogenesis and DNA damage.

It is difficult to establish a temporal hierarchy of these events and to determine which is first. We are faced with intricate mechanisms that influence each other, and all are potential candidates to be the final “trigger” of cancer. It has been suggested that “metabolic reprogramming” could be a hallmark of metabolism itself and not specific to cancer cells [36]. If metabolic adaptive capacity is inherent in all cells, the specificity of tumor cells might lie in the enhanced ability to do so (by over-expressing potentially pre-existing processes) [13]. Certain behaviors may also never appear for normal cells because the environmental conditions necessary for the appearance of these phenotypes do not occur under physiological conditions. It is not unreasonable to consider metabolic transitions without genetic alteration [37].

**Figure 1:**
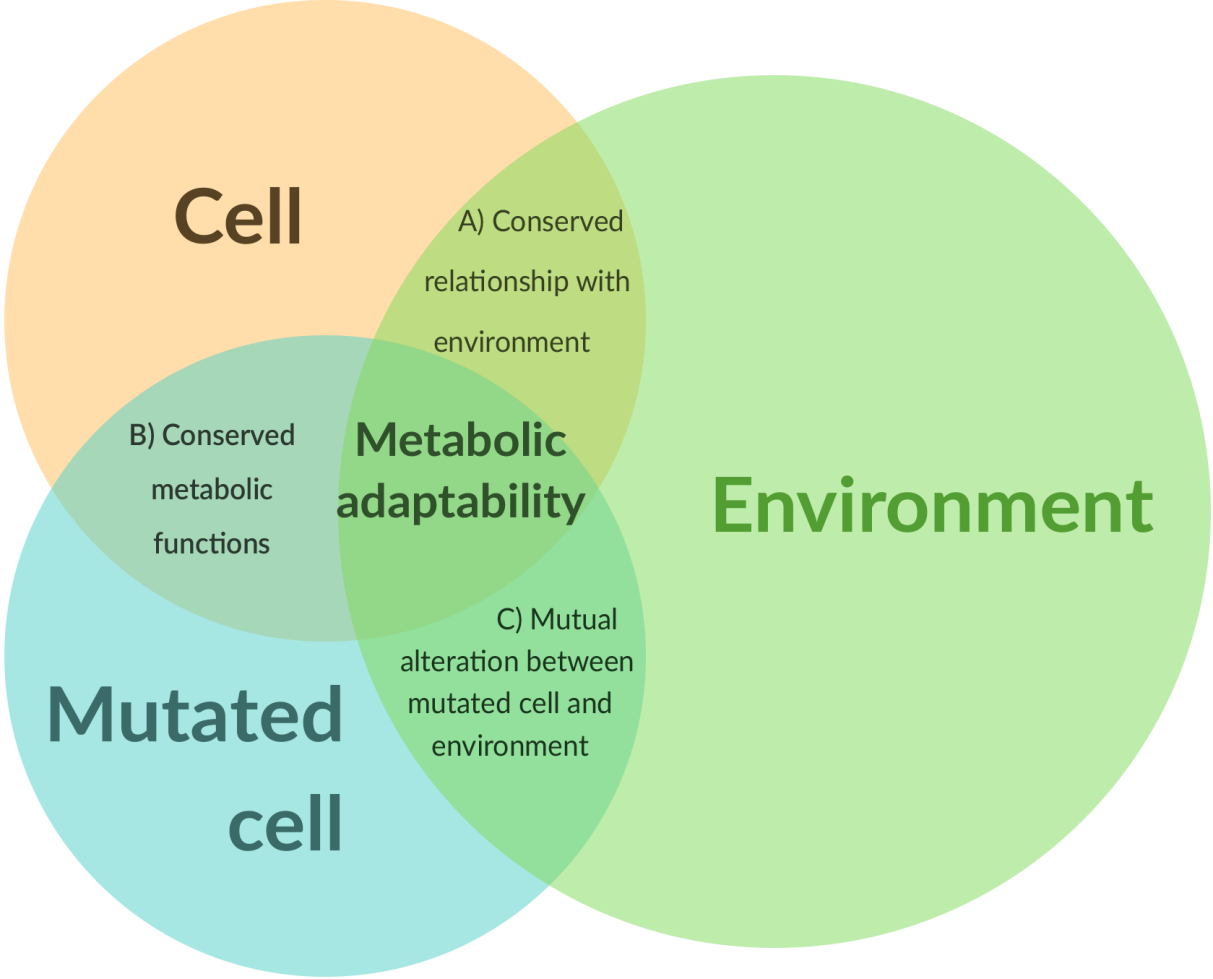
Schematic representation of metabolic adaptability. Metabolic adaptability lies at the interface of the relationship between the cell’s structure and its environment, and is beyond only mutations.

This metabolic adaptability should be expressed on several levels of interactions. Intact cells (A in Figure 1) interact with the environment through biochemical processes (secretions, uptakes), and the thermodynamic properties of the enzymes involved allow a first level of regulation. Epigenetics offers a second level of regulation, which adjusts the cell–environment relationship within a (context-dependent) acceptable limit for the cell and the surrounding ones and allows the structural integrity of the environment to be maintained. Some metabolic functions (if not most) are conserved upon mutations (B in Figure 1), and the majority of the regulatory mechanisms that are valid for intact cells are also valid for mutated cells. However, in the context of a mutated cell (C in Figure 1), the cell–environment relationship can, however, be drastically altered. These mutations can increase the expression of ion channels [38] or transporters on the surface of the cell, thus modifying the dynamics of exchanges. Conversely, an altered environment can itself promote mutations within the cell. Metabolic adaptability emerges as a common property of intact and mutated cells in relation to their microenvironment. The notion of “adaptability” is not specific to a particular cell type, even if this capacity can be more or less favored within a specific cell–environment duo.

The prevalence of certain states within the metabolic landscape may indicate the presence of attractors without ruling out the possibility of escape. The existence of these attractors could depend on the structure of the cell, as well as on the underlying thermodynamic, biochemical, or biomechanical mechanisms. A recent model of metabolism has been proposed to study its landscapes [39]. The authors described the presence of attractors in large dimensions and showed the possibility of transit from one to others depending on cellular and environmental conditions. According to the authors, a “switch”, as discussed, can be observed in cancer cells, but is the consequence of “non-equilibrium dynamics and thermodynamics” in specific conditions, rather than a direct emergence from the biological perspective.

## 5. Conclusions

Metabolic reprogramming is a concept frequently used to refer to the metabolic transitions that can take place within tumor cells. This notion seems conceptually well understood because it echoes the computer culture and is sufficient to illustrate a large part of the observed processes. This poses, however, a first semantic problem, which is to involuntarily give cancer cells an anthropomorphic behavior—a cell able to modify its own code by itself for a specific purpose. This perhaps contributes to the encouragement of the community to focus on the study of tumors at the genetic/epigenetic level at the expense of other levels of molecular interactions, although that tends to change with the rise of new omics [36]. Trans-omics [40], in particular, could be encouraged in the future in order to integrate these different levels of interactions. The second problem is that of clarifying the mechanisms underlying this “reprogramming”. Heterogeneity must be taken into account when the metabolism of a tumor is studied. This makes the identification of key metabolic features difficult. So, more importantly than identifying these features, identifying “cell states” in relation to the environment can help define a metabolic landscape in which cells can transit [39]. It might also be appropriate to distinguish, on the one hand, a cell metabolism, and on the other, a tissue-level metabolism/phenotype. As a tissue, a tumor can present a Warburgian phenotype with a high consumption of glucose and production of lactate, but not necessarily everywhere and all the time at the cell level [13]. Finally, it may be necessary to reintegrate the capacity for the cell to transit within this metabolic landscape, adopting several of these states depending on environmental conditions, by considering its metabolic adaptability, i.e., the set of molecular mechanisms achievable by the cell with or without mutation that allow it to survive in its environment. Can we finally think of the metabolism in a less genocentric manner [36] to include, as the opposite, all of its levels of complexity in a more holistic framework?

## Data Availability

Not applicable.
